# Frontotemporal network interactions causally support rapid concreteness judgments during reading

**DOI:** 10.1371/journal.pbio.3003723

**Published:** 2026-03-25

**Authors:** Elliot Murphy, Oscar Woolnough, Cale W. Morse, Xavier Scherschligt, Nitin Tandon

**Affiliations:** 1 Vivian L. Smith Department of Neurosurgery, McGovern Medical School, University of Texas Health Science Center at Houston, Houston, Texas, United States of America; 2 Texas Institute for Restorative Neurotechnologies, University of Texas Health Science Center at Houston, Houston, Texas, United States of America; 3 Memorial Hermann Hospital, Texas Medical Center, Houston, Texas, United States of America; New York University, UNITED STATES OF AMERICA

## Abstract

Neurobiological models of conceptual processing have been limited in spatiotemporal resolution, and uncertainty remains about the causal role of specific regions in concept representation. We utilized intracranial recordings in human neurosurgical patients with epilepsy (*n* = 19) during a concreteness judgement paradigm of single word reading. Concrete concepts showed greater high-frequency activation across a frontal and ventrotemporal network, while greater activation for abstract words was found in lateral posterior middle temporal cortex. Intercortical communications, measured by high-frequency partial direct coherence, revealed bidirectional frontal and ventral plus lateral temporal interactions. Words occupying the middle range of the concreteness scale (e.g., “profit”) activated similar regions, but high-frequency signatures were not modulated by the participants’ semantic decisions about these words. Cortical stimulation of ventrotemporal cortex and inferior frontal cortex disrupted the ability to make concreteness judgements. These results suggest that semantic information is encoded via a causally directed system of bidirectional cortical cascades: early visual-linguistic integration in ventrotemporal cortex initiates directed information flow to frontal hubs, and later processing shows reciprocal flow back to ventral and lateral temporal regions integrating distinct conceptual features, with these convergence zones differing based on semantic type. Our results provide a systems-level account for how the human brain transforms word forms into grounded conceptual meaning that is invariant of subjective judgment.

## Abstract 2

How the brain causally represents conceptual meaning with precise spatiotemporal dynamics remains unclear. This study shows through brain stimulation and intracranial recordings that concrete and abstract words engage frontotemporal networks, with bidirectional cortical interactions and causal hubs supporting a cascade that transforms visual word forms into representations.

## Introduction

A fundamental human cognitive attribute is the representation of concepts that can be either concrete or abstract. While many animals can refer to concrete objects [1,2], the ability to assimilate and generalize abstract concepts appears exceedingly sparse [3]. The attributes of concrete, perceptible concepts (e.g., *table*, *hammer*) and abstract, imperceptible ones (e.g., *justice*, *decision*) [4] is present across word classes (adjectives: *red/sad*; nouns: *table/justice*; verbs: *eating/thinking*; adverbs: *gently/happily*). Concrete words are easier to learn [5], read [6], remember [7,8] and define [9].

Understanding how the cortex distinguishes between abstract and concrete concepts critical to elucidating the broader systems-level mechanisms via which the brain constructs rapid lexico-semantic inferences (e.g., inferring whether a word refers to a concept that has objecthood, concreteness, and a conceptual feature structure) [10–13]. Recently, other causal processes outside of commonly considered neural mechanisms are being entertained as providing explanatory and predictive power in the cognitive neurosciences [14,15]. For instance, research on cortical cascades is becoming increasingly relevant to understanding neurological disorders and developing new diagnostic and therapeutic approaches to cognitive deficits [16–19], but how such causal structures are leveraged at the whole-brain level during lexico-semantic processing remains incompletely addressed in the literature. These cascades refer to temporally ordered, causally coupled sequences of neural activations that propagate across distributed cortical regions, whereby early activity in one area constrains and shapes subsequent processing in downstream regions. Recent accounts of causation in neuroscience call for acute consideration of temporal dimensions to better isolate causal-mechanistic processes [14], which is difficult to address through non-invasive recording modalities. Invasive recordings can offer higher spatiotemporal resolution in addition to causal evidence via electrical stimulation [12,20].

There remains considerable controversy over which brain regions are implicated in the concrete/abstract word distinction, due largely to the multiple methodologies used and wide differences in task complexity. Contradictory roles have been assigned to the anterior temporal lobe, implicating it in both abstract [21,22] and concrete-selectivity [23,24]. These dueling perspectives can arguably be reconciled by assuming its role in multiple semantic demands, whereby meaning is reconstructed in conjunction with multiple network nodes connected to the anterior temporal lobe [25]. Certain regions seem to have their activity modulated not exclusively by conceptual category, but by task demands, stimulus structure, and whether neural recordings are sensitive to early feedforward versus feedback dynamics [26,27]. Functional imaging implicates the inferior frontal gyrus (IFG) and middle temporal gyrus (MTG) in abstract word processing [28–32], while concrete concepts have been shown to activate left posterior cingulate, precuneus, fusiform gyrus, and parahippocampal cortex [22]. The left IFG has been implicated in abstract word processing [28], concrete word processing [33], and both [34]. In addition, compilations of concrete/abstract processing in meta-analyses cannot disambiguate single-word from sentence-level effects. Further complicating this issue, many word stimuli utilized in concreteness studies occupy a space somewhere “between” concreteness and abstractness [35–41]. For example, “magic,” “profit,“ and “corporation” can all be read as simultaneously hosting a mixture of concrete and abstract features.

To help resolve these inconsistencies, we used intracranial recordings in 19 neurosurgical patients with epilepsy while they performed a single-word concreteness judgement task. Additionally, we causally mapped disruptions of concreteness judgements by delivering targeted electrical stimulation in five of these patients. We focused on semantic processing from single visual word presentations of concrete words, abstract words, and what we termed “midscale” words. Concrete, abstract and midscale stimuli were taken from a previous study that carefully selected items based on their concreteness ratings from a number of independent databases [42,43]. These midscale words display mean concreteness ratings occupying the middle range of the 1–5 Likert scale and exhibit larger standard deviations (i.e., exhibit greater inter-individual disagreement, leading to the mean occupying the middle of the scale) [42]. The inclusion of midscale words allowed us to assess the processing of words that can potentially be perceived as either concrete or abstract (e.g., “profit” can saliently trigger a sense of physical money, or an abstract and stable means of exchange). Our goal was to address controversies in the literature concerning (i) the neural representation of concreteness (i.e., the structured pattern of neural activity and connectivity that reliably encodes task-relevant information), not just its cortical localization, (ii) the neurobiology of the processing of midscale words that occupy a semantic space lacking a simplistic semantic classification, and (iii) the causal involvement of specific brain regions in these word classes.

## Results

**Fig 1:**
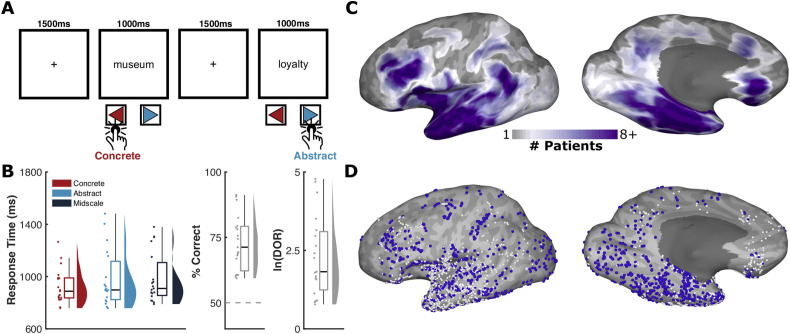
Experimental paradigm and patient coverage. **(A)** Schematic representation of the concreteness judgement task. **(B)** Behavioral performance of patients, showing mean response times for each word class, percentage correct responses and each patients’ log diagnostic odds ratio (ln(DOR)). All included patients had a significant ln(DOR). **(C)** Representative coverage map (19 patients) and (D) individual electrode locations (2,241 electrodes) for the left hemisphere, highlighting electrodes responsive over baseline in blue (1,030 electrodes; BGA > 10% in the time window 300–700ms after word onset). White spheres indicate inactive electrodes. Source data for these results can be found at OSF (osf.io/jyvg2/).

Nineteen native English-speaking patients participated in the experiment (6 male, 19–47 years, all right-handed, IQ 96 ± 8, Age of Epilepsy Onset 20 ± 11 years). All participants were semi-chronically implanted with intracranial electrodes for seizure localization of pharmaco-resistant epilepsy. Patients were presented with single words on a screen and asked to press a button indicating whether the word referred to something they could directly perceive (Fig 1A). We used broadband gamma activity (BGA; 70–150 Hz) as a measure of local cortical processing [44–47].

### Behavioral analysis

Participants provided a response for 98 ± 2% of trials. Mean classification accuracy was 67 ± 12% for concrete words and 77 ± 15% for abstract words. Participants’ mean log diagnostic odds ratio (ln(DOR)) was 2.2 ± 1.2 with all included participants performing significantly above chance. Participants rated 65 ± 18% of midscale words as abstract. Mean response times for each category were: 932 ± 134 ms (Concrete), 986 ± 221 ms (Abstract), and 989 ± 198 ms (Midscale) after word onset. We suspect that the inherent difficulty of the task, and its semantically demanding nature, in addition to the inclusion of the “midscale” possibility at any moment (which primes an additional level of semantic engagement from participants), likely explains the reported accuracy scores. Nevertheless, the number of correct trials in this study still matched or exceeded prior well-powered intracranial reports [48,49].

### Spatiotemporal correlates of concreteness

**Fig 2:**
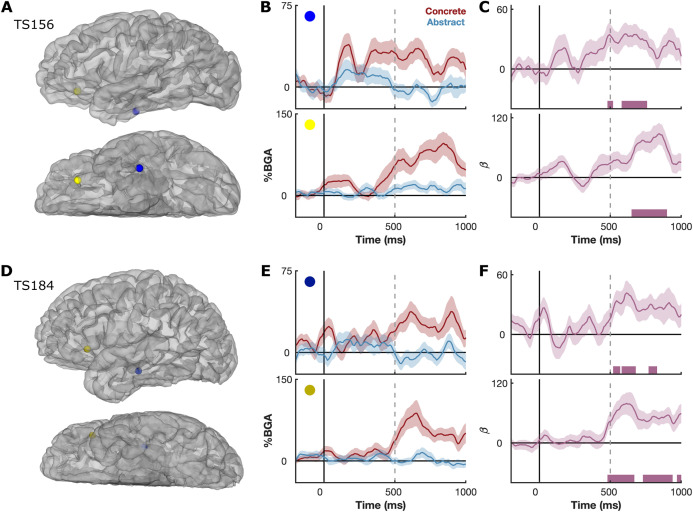
Single patient responses to concrete and abstract words. **(A,D)** Location of recording electrodes in individual patient space. **(B,E)** Mean BGA (± SE) for concrete and abstract words, and **(C,F)** LME beta values (*β* ± SE) for the concreteness regressor. Word length and frequency effects were regressed out of the LME. Colored bars represent regions of significance from the LME analyses (*q* < 0.01). Results shown for patients TS156 **(A–C)** and TS184 **(D–F)**.

Distinctions between concrete and abstract words were discernible at the level of single electrodes, with concrete words showing greater activity than abstract words across numerous sites. We show data from two representative patients with simultaneous coverage in ventrotemporal and lateral frontal cortex (Fig 2). Both patients show clear distinctions between concrete and abstract words in both regions, beginning around 500ms after stimulus onset.

**Fig 3:**
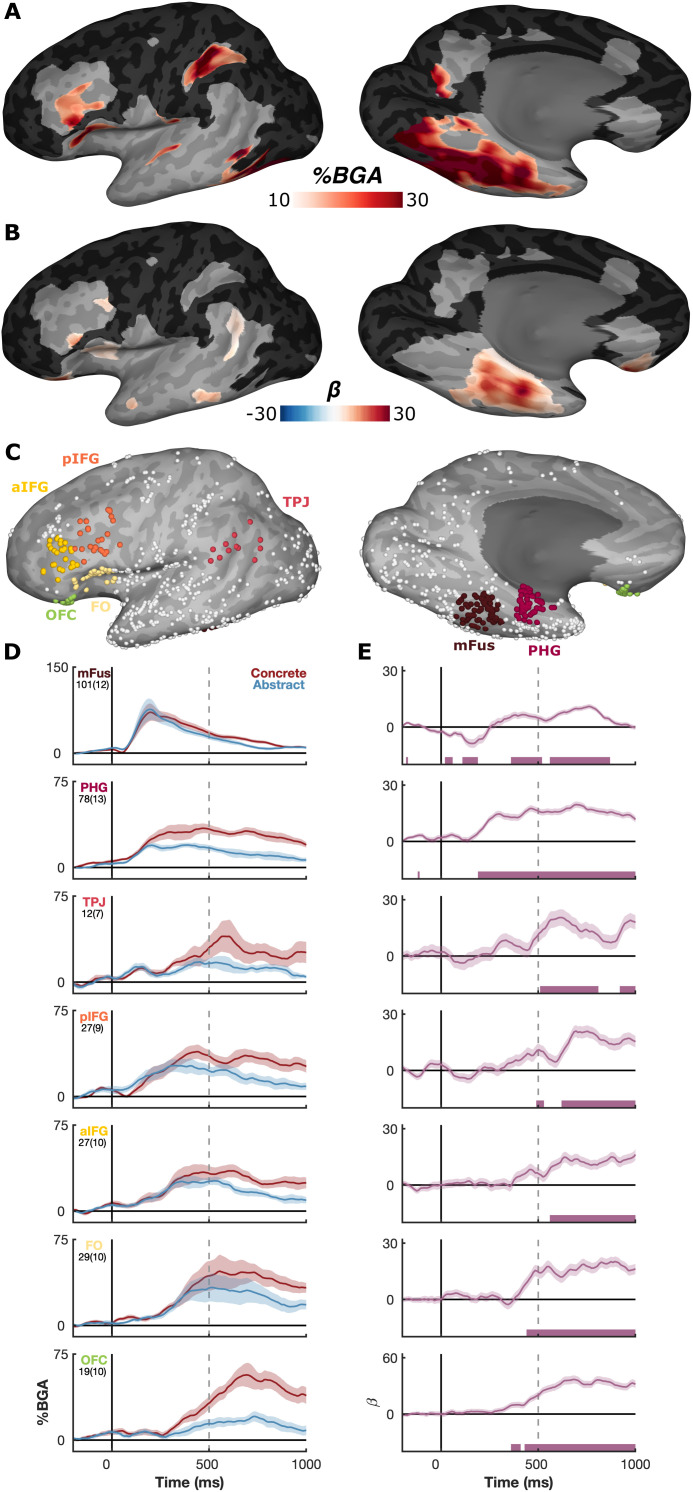
Spatiotemporal neural correlates of concreteness. **(A)** Activation above baseline during the task for all stimuli types (300–700 ms). **(B)** Concreteness regressor from the surface-based linear mixed effects (sbLME) model, showing significant clusters (*t* > 2.5, coverage > 2 patients, *p* < 0.01 corrected). **(C)** Locations of electrodes included in each functional ROI. **(D)** Mean BGA (± SE) for concrete and abstract words, and **(E)** LME beta values (*β* ± SE) for the concreteness regressor. Word length and frequency effects were regressed out of the LME. Number of electrodes and patients per ROI are shown below each ROI label as “electrodes(patients).” Colored bars represent regions of significance from the LME analyses (*q* < 0.01).

To investigate the spatial extent of effects across individuals we implemented a surface-based linear mixed effects (sbLME) model [50] for the window 300–700 ms after stimulus onset, a period we have previously demonstrated as crucial for semantic processing of written words [51]. This allowed us to combine responses across individuals in a standardized cortical space, isolating effects of concreteness and regressing out confounders such as word length and frequency. This analysis highlighted a broad concreteness cluster across ventrotemporal cortex, extending from mid-fusiform to anterior fusiform gyrus. We observed significant clusters across lateral inferior frontal cortex, orbitofrontal cortex (OFC), and temporoparietal junction (Fig 3B), many of which also showed general task-related activity (Fig 3A). A lower threshold analysis revealed that lateral posterior middle temporal cortex showed significantly greater activation for abstract over concrete words (S3 Fig).

We further investigated the temporal profile of these distinctions across ROIs in these regions (Fig 3C). The earliest regions where we observed reliable differences between concrete and abstract words were parahippocampal gyrus (PHG) and mid-fusiform cortex (mFus) at approximately 250 ms after word onset (Fig 3D and 3E). Effects in frontal regions were found from approximately 400ms onwards, beginning in OFC, then frontal operculum (FO), and finally anterior followed by posterior IFG. Lastly, posterior temporoparietal cortex (TPJ) showed an effect at 500 ms. These regions all showed greater BGA for concrete over abstract words. All effects lasted into the 1,000 ms mark, with the exception of mFus, which was bounded to the 400–800 ms window.

**Fig 4:**
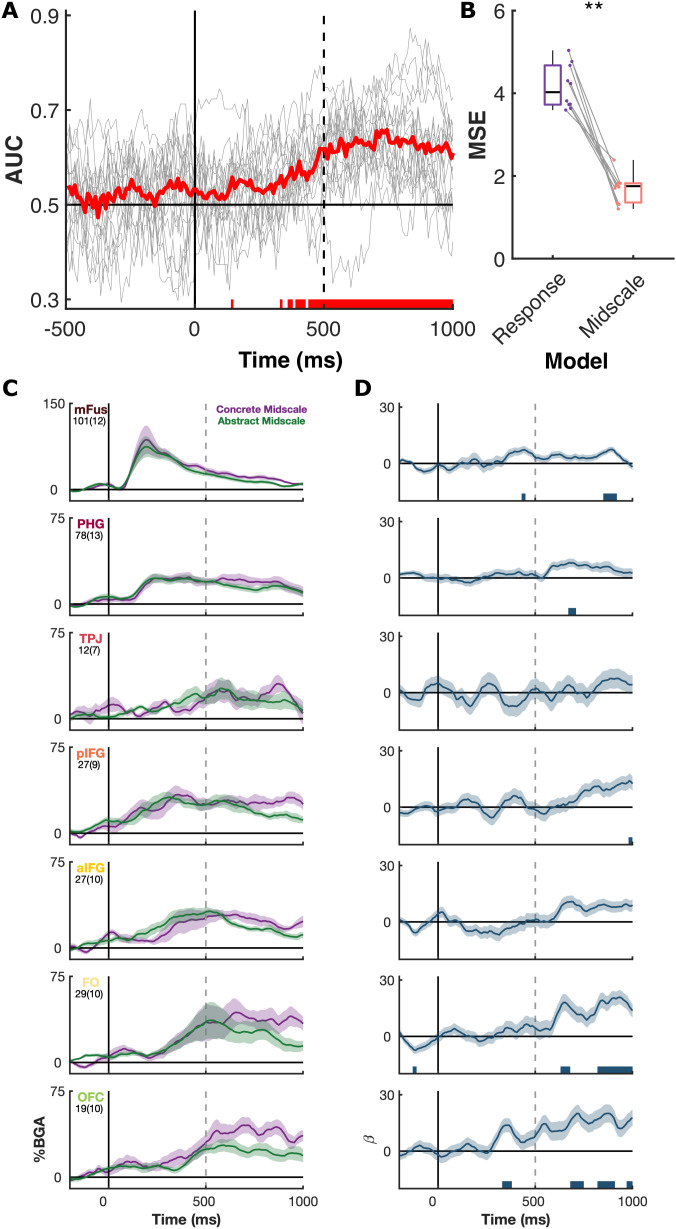
Neural responses to midscale words. **(A)** Mean single-trial decoding results (red), represented as area under the curve (AUC), from linear support vector machines (SVMs) trained within each patient (gray lines) to distinguish concreteness over time. **(B)** Mean squared error (MSE) of support vector regressors trained on concrete vs. abstract word responses, in the window 500 to 800ms, and tested on the midscale words, comparing two competing models. ** *p* < 0.01. **(C)** Mean BGA (± SE) for concrete and abstract rated midscale words, and **(D)** LME beta values (*β* ± SE) for the regressor contrasting these conditions. Word length and frequency effects were regressed out of the LME. Number of electrodes and patients per ROI is shown as (electrodes(patients)). Colored bars represent regions of significance from the LME analyses (*q* < 0.01). Source data for these results can be found at OSF (osf.io/jyvg2/).

To further characterize the whole brain timescale of concreteness processing and investigate the reliability of single-trial representations, we utilized a linear support vector machine (SVM) decoder to distinguish concrete and abstract trials based on the time course of their BGA (Fig 4A). This decoder displayed high accuracy (peak median accuracy: 66%), with 13 patients showing >70% accuracy (peak individual accuracy: 87%). Decoding accuracy across patients reached significance at ~360 ms and was maximal around 650 ms.

### Neural representation of midscale words

Based on these results, we trained a decoder on the time window of 500–800 ms after word onset to predict patients’ responses to the midscale words. Ten patients showed significantly greater predictive ability than their null distributions when deciding between concrete and abstract words (group-level Wilcoxon sign rank, *p* < 0.001, ln(BF_10_) = 4.7). Support vector regression (SVR) decoders were trained based on the concrete (concreteness = 5) and abstract (concreteness = 1) trials in these patients and were then tested on the midscale trials. We tested the results against two competing models: (i) a response-based model where the expected concreteness was based on the participants’ ratings (concrete = 5, abstract = 1), and (ii) a midscale model predicting that all the midscale trials should fall between concrete and abstract (concreteness = 3), regardless of how they were rated by participants. In all 10 patients we observed a lower mean squared error (indicating a better model fit) for the midscale model (*p* = 0.002, ln(BF_10_) = 8.5) (Fig 4B). Note that we discuss some inherent limitations to this analysis in our Discussion section (see “Limitations,” below) concerning an intrinsic variance asymmetry between the two competing label sets.

When we contrasted neural responses to midscale trials rated as concrete versus those rated as abstract, within the ROIs used previously we did not observe reliable differences in BGA (Fig 4C and 4D). Some effects were observed in FO and OFC at late processing stages, although these were transient. We did not observe any specific effects of midscale words, distinct from showing an intermediary response between that of concrete and abstract words (S2 Fig).

### Semantic network connectivity

**Fig 5:**
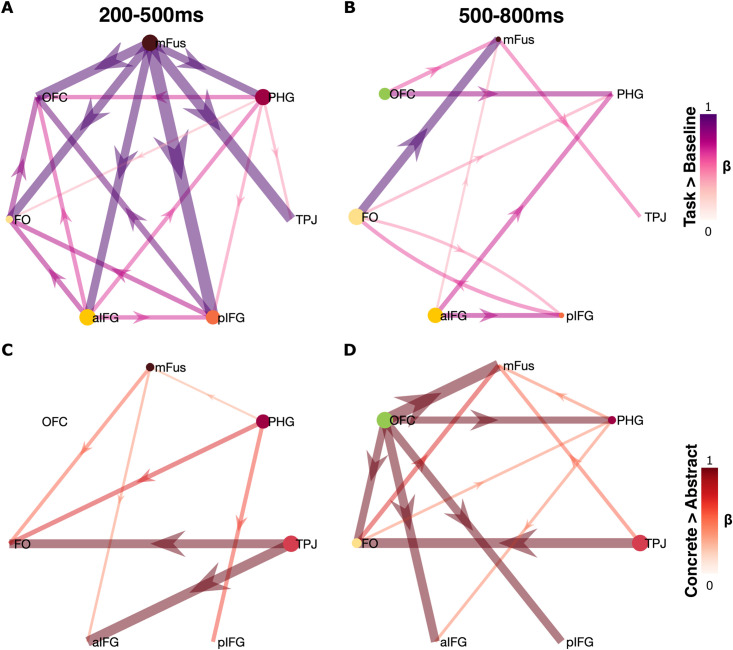
Connectivity across the semantic network. Partial directed coherence (PDC) network connectivity map between all seven ROIs for **(A,B)** the overall PDC and **(C,D)** the increase in PDC for concrete words greater than abstract words. Arrow color represents the mean PDC strength between regions, in the windows 200–500 ms **(A,C)**, and 500–800 ms **(B,D)** post-stimulus onset. Node size for each ROI is scaled by the magnitude of outflow from the ROI. Arrows are shown for significant connections (FDR corrected, *q* < 0.05). Source data for these results can be found at OSF (osf.io/jyvg2/).

To quantify the spread of information within the semantic network during word processing, we used partial directed coherence (PDC) within BGA to quantify directional inter-regional interactions. In an early time window (200–500 ms), net information flow occurred primarily from mid-fusiform to lateral temporal to frontal cortices, but also from anterior IFG to parahippocampal cortex (Fig 5A), whereas in a later time window (500–800 ms) information flows were primarily from frontal cortices to ventrotemporal hubs (Fig 5B). During processing of concrete words, in the early window net information flow was greater from ventral and lateral temporal cortex to frontal cortex for concrete words (Fig 5C). In the later window, OFC was a source hub, with greater information flow to other nodes across the network during concrete word processing (Fig 5D). TPJ showed greater output to FO during concrete word processing through both time windows.

### Causal involvement in concreteness processing

**Fig 6:**
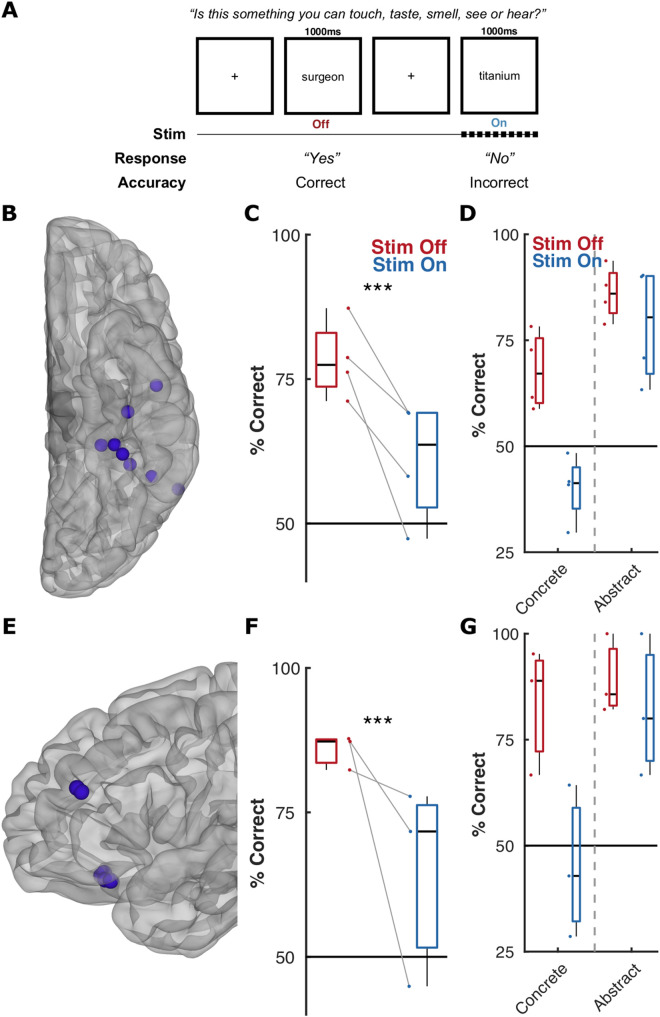
Stimulation of ventrotemporal and frontal cortex disrupts concreteness judgements. **(A)** Schematic representation of the concreteness stimulation task. No midscale words were used, only concrete and abstract words that patients had previously accurately identified during the prior intracranial task. **(B,E)** Locations of the stimulated pairs on a standardized brain surface for (B) ventrotemporal and (E) frontal sites. **(C,F)** Mean overall accuracy, and (D,G) mean accuracy split by concrete or abstract words, during each stimulation session during trials with stimulation off (red) or on (blue). *** *p* < 0.001; binomial GLMM. Source data for these results can be found at OSF (osf.io/jyvg2/).

Thus far, we have implied regional involvement in concreteness processing from intracranial activation data. Moving beyond this, to test the causal role of ventrotemporal cortex and frontal cortex in concreteness judgements, we performed direct electrical cortical stimulation in five participants, at four separate sites across the ventral surface and three sites across frontal cortex. These were selected for further study based on significant activation distinctions between concrete and abstract words. Participants performed a concreteness judgement task, using only concrete and abstract words they had correctly assigned previously in the main task (excluding all midscale words), while a random 50% of the words (equal across both conditions) were accompanied by stimulation (Fig 6A). All stimulation sessions occurred at least one day after the initial concreteness experiment reported above, and no more than six days after. All tested sites resulted in disruption of narrative reading during clinical stimulation, indexing engagement in the reading network. Stimulation within ventrotemporal cortex led to a universal disruption of concreteness judgements, with stimulation of all four sites resulting in a reduction of concreteness judgement accuracy (binomial GLMM, *t*(418) = −3.5, *β* = −0.81, *p* < 0.001, 95% CI −1.27 to −0.35) (Fig 6C). We did not observe a significant interaction effect on accuracy between stimulation and whether the word was concrete or abstract (*t*(418) = −1.4, *β* = −0.64, *p* = 0.17, 95% CI −1.56 to 0.28). While we note that these effects may be inevitably due to some level of general alexia, not specific to semantic processing, we also wish to highlight that patients mostly provided a Yes/No answer.

Within frontal cortex we also observed significant deficits in the ability of the participants to accurately distinguish concrete and abstract words (*t*(237) = −3.9, *β* = −1.31, *p* < 0.001, 95% CI −1.98 to −0.64), however, here we also observed greater disruption of the ability to correctly identify concrete words compared to abstract words (*t*(237) = −2.3, *β* = −1.60, *p* = 0.019, 95% CI −2.95 to −0.26). A comparison with the above analyses (Fig 5) indicates that mFus and inferior frontal sites are the major, critical nodes in the semantic network (i.e., regions that host salient semantic information, within the context of a broader, connected network) pertinent to concreteness.

**Fig 7:**
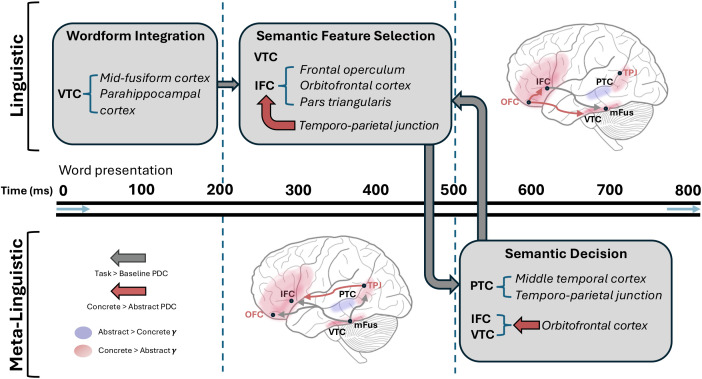
Schema of main effects of concreteness across multiple processing windows. Segregating into (broadly) linguistic (e.g., lexico-semantic) and meta-linguistic (e.g., executive decision-making) stages across the temporal windows analyzed in our PDC (200–500 ms, 500–800 ms) and high-frequency activity plots. Regions of interest depicted are chosen based on their occurrence across multiple analysis windows and types of statistical tests: ventrotemporal cortex (VTC), inferior frontal cortex (IFC), posterior temporal cortex (PTC), orbitofrontal cortex (OFC), mid-fusiform cortex (mFus), and temporo-parietal cortex (TPJ) (see Figs 3 and 5). Colored brain regions (red: concrete; blue: abstract) denote which category showed the greatest increase from baseline (see S3 Fig).

We provide a summary of our main findings (Fig 7) that captures the most salient effects from across our set of results.

## Discussion

We uncovered widespread frontotemporal pathways that are engaged in making semantic assessments, anchored to information outflow from ventral occipitotemporal cortex to inferior frontal cortex. We provide mechanistic insights via causal evidence (direct cortical stimulation) and interactional connectivity dynamics charting rapid semantic decisions about concreteness across a broad cortical map. These results suggest that semantic decisions pertaining to concreteness are indexed via an information routing mechanism whereby lexico-semantic features relevant to concreteness are accessed. One possible lens to capture these results is to consider this process as a series of cascades with distinct neural interactions: early visual-linguistic integration between ventrotemporal cortex (i.e., the triggering from sensory information specific stored linguistic items, such as morphemes) and frontal convergence hubs serves to anchor semantic representations to perceptual feature information. Our analyses also revealed rapid frontal feedback to ventrotemporal cortex within our early processing window. We consider these “hubs” to be highly connected cortical regions that integrate information from multiple distributed sources. These frontal hubs are sensitive to salient semantic features of lexical items, and interact with (concreteness-relevant) ventrotemporal hubs to derive semantic information. We discuss below how our results contribute to a network-level account for how the human brain transforms word forms into grounded conceptual meaning, differing from existing models of representational integration.

Ambiguous stimuli that do not occupy a clear space on the concreteness scale (midscale words like “district,” “patriot,” “expanse,” “asset,” “gossip”) revealed an invariant activation profile primarily sensitive to conceptual classification. The documented neural responses to midscale words (Fig 4) suggest that broadband high gamma activity serves as a reliable index of higher-order semantic properties (that help determine whether a word denotes primarily abstract or concrete features) that do not reduce simply to direct task demands. Direct cortical stimulation shows the causal involvement of ventrotemporal and frontal cortex in making concreteness inferences. To assess if any cortical regions show greater activity for abstract concepts, we used a less conservative statistical threshold for the population mixed effects analysis, and also analyzed effects at the single electrode level. The vast majority of regions and electrodes were more active for concrete concepts, but portions of STG and pMTG showed greater engagement for abstract over concrete words (S3 Fig). These findings have broader implications for cortical computation, suggesting that meaning is instantiated in a robust series of frontotemporal cascades, independent of subjective introspection.

### Cortical cascades for conceptual processing

Cognitive neuroscientists are tasked with offering relevant causal explanations suited to their methodological constraints and phenomena of interest. These are sometimes classically mechanistic, but the full causal landscape of the brain likely admits for multiple types of causal concepts (relating to pathways, topological structures, non-causal mathematical models, and notions from dynamical systems theory) [14,15]. For instance, mechanisms involve part-whole constitutive relations; cascades do not. Instead, cascades have causal influence via “relationships between distinct” factors [52]. In biology, there are blood coagulation cascades, cell signaling cascades, and other related causal structures that involve an initial trigger, sequential amplification, and a stable convergence to a final state [53,54]. For example, Damasio’s classic theory of semantic recall explicitly invokes cascades directed from the anterior temporal lobe as a core explanatory structure [25], and Coltheart and colleagues invoke a dual route cascaded model for visual word reading [55]. Within the neurosciences, cascades do not have to be strictly serial, and readily admit for dynamic, partially ordered, and recurrent processes that can support bidirectional interactions.

We suggest that there are particular dynamics for cortical cascades can account for our documented causal and interactional dynamics, accommodating for the flow, direction, and timing of dynamics subserving basic conceptual processing. Cortical cascades have been shown to provide robust causal structure in systems neuroscience [11,18]. Within the context of our results, ventrotemporal regions may host conceptual features accessible via graded sensory salience [56,57]. Local gamma increases here represent recurrent excitation seemingly stronger for concrete concepts. The precise timing of these gamma increases initiate cascade dynamics by triggering the outflow of information from ventrotemporal sites. Afterwards, hierarchical propagation to frontotemporal convergence hubs is indexed in our results by directed connectivity from temporal to frontal sites showing content-sensitive information transfer. These include, but are not limited to, language-selective integration hubs [10,58,59]. Next, salience-based integration is subserved by these frontal sites. For instance, OFC and FO may implement a semantic salience map [60–62], integrating multimodal features to evaluate whether a representation has sufficient sensory grounding to be “concrete.” The stronger effect of stimulation on concrete word judgments in frontal cortex supports this specialization. These frontal sites may provide the semantic gating signal needed to finalize judgments. Lastly, the invariant encoding of conceptual features speaks to a robust and generalizable set of neural dynamics that apply across all word classes. Later re-entrant activity supports further refinement via feedback to ventral and lateral temporal sites. Our midscale word results imply a conceptual representational core that is (1) robust to task demands; (2) not entirely derived from general decision-making; and (3) possibly grounded in population-level neural codes optimized for feature convergence (e.g., via attractor dynamics) [63,64]. This frontotemporal semantic network operates through directional, frequency-specific, and temporally sequenced dynamics, where gamma activity reports a semantic convergence signal. Causal disruption of cascading information flow prevents the network from accumulating sufficient evidence for semantic classification.

Critically, our model can accommodate bifurcating cascade routes that direct concepts of distinct featural types. For instance, our results indicate that concrete concepts activate ventral temporal areas that initiate bottom-up cascading towards frontal sites, whilst simultaneously anterior frontal cortical sites provide early top-down information to these ventral temporal sites to semantically contextualize orthographic-to-lexical transformations. In contrast, abstract concepts, lacking strong perceptual anchoring, recruit lateral posterior temporal areas early and then cascade forward to semantic control hubs in frontal cortex. Importantly, concrete concepts also exhibit this frontotemporal dynamic, supporting our claim that the sites of semantic convergence remain frontal for all word types, including midscale words. Multiple possible cascade routes feed into frontal convergence zones but may engage distinct convergence sub-circuits depending on semantic type. For instance, after cascading from mFus to a broad set of frontotemporal regions (Fig 5A), information appears to be sent from OFC to distinct convergence sites across broad portions of frontal cortex (Fig 5D). Our cortical stimulation results acutely support this general architecture (Fig 6), in accord with interventionist theories in philosophy of biology [65], with an eye towards causal explanation [66,67].

As we note below, existing models of conceptual processing do not offer clear mechanistic candidates (e.g., hub-and-spoke models typically provide only an atemporal schema for which brain regions may process semantic information; but see [27]), and they make predictions for the role of specific regions that may fall into neuroanatomical overcommitments, e.g., in many models anterior temporal lobe is seen as the sole “hub” integrating distinct concepts [68]; meanwhile, embodied cognition accounts would expect a sharper alignment between cortical responses to concreteness and subjective rating). While many hub-and-spoke and embodied models lack specificity in this regard, our results identify specific temporal windows and directional flows of high-frequency activity as a basis for accessing conceptual features and integrating them into a coherent semantic decision. Early processing flows from ventrotemporal to frontal sites, and later processing shows reciprocal flow from frontal back to ventral regions. This two-phase dynamic is not accommodated in classical hub-based or embodiment accounts: we did not find a singular hub, but rather multiple streams that converged over multiple hubs (mFus, OFC) modulated by the semantic features in question. This dynamic is readily captured by a cascade model since the amplification of information flow is modulated by features such as semantic representation type, mirroring other cascade structures in biology [18]. With respect to possible time course commitments of hub-and-spoke architectures, we note that our results seem to align with the work of Rogers and colleagues [27] that predicts multiple “waves” of activity (early and late) in ventral anterior temporal sites, where we see early sensitivity to concreteness followed by later information flows.

### Cortical hubs of concreteness

There are also alternative, plausible explanations for our results, that do not rely on the types of theoretical structures discussed thus far.

For example, our results could be interpreted as being orchestrated by a dynamic hub-and-spoke system, rather than a cascade per se. This is rendered plausible given that mFus and inferior frontal cortex both show hub-like properties [27]. In addition, directed connectivity does not by any means exclusively imply cascades. As such, while we note again that our specific combination of *directionality*, *timing* and *causal disruption* speaks to the presence of behaviorally relevant cortical cascades for semantic processing, we stress that our results are also compatible with temporally extended hub-and-spoke architectures in which multiple hubs engage sequentially, rather than simultaneously, as already envisioned in prior models [25,26].

In addition, while we regress out word length and frequency effects, and while our documented effects persist long after initial word presentation, we can nevertheless not fully exclude the possibility that some ventrotemporal effects reflect properties of the reading network itself, rather than semantic processing (for further psycholinguistic details about reading, see also [55,69]).

Another explanation comes from some rather elementary observations about the nature of concrete concepts, already noted above. Consider the possibility that concrete words may simply evoke stronger, more coherent neural signals, rather than qualitatively different representational formats. Concrete words are acquired earlier in life than abstract words, have higher imageability, and are more consistently represented across individuals. Meanwhile, high-frequency gamma power scales with local population synchrony: gamma activity does not directly implement specific computations, but instead seems to constitute an activity motif that reports the cellular substrates, communication channels, and computational processes underwriting information processing [70]. Thus, any differences documented in intracranial recordings between concrete and abstract words may reflect graded representational strength or signal-to-noise differences rather than distinct representational architectures. Consider in this context our discovery about midscale words, and how they show intermediate (but stable) activation. This supports theories that invoke graded representational strength rather than categorical semantics – which, indeed, also mirrors recent trends in models of semantics [13,39,61,71].

A general finding of neuroimaging studies investigating concreteness [31] is that the left IFG and MTG appear to act in concert to organize abstract word processing across lexical classes [28–30,32]. This appears to more broadly reflect the organization of the frontotemporal language network [50].

With respect to other current models, the Dual Coding theory postulates that concrete words are supported by both perceptual and verbal representations, while abstract words are solely based on linguistic information, such that their semantic content is *relational* and depends on domain-specific, language-internal structure [33,72]. The posterior middle temporal gyrus (pMTG), that we have previously implicated using intracranial studies as a core higher-order language region [49,50,62,73], showed greater engagement for abstract words, providing some support for these theories (S3 Fig). This portion of pMTG—being implicated in basic syntactic, semantic, and (abstract) conceptual processing—overlaps with portions of the MTG that show human-unique extensions in the arcuate fasciculus [74], potentially underwriting its species-defining role in structured symbolic abstraction. Indeed, other individual electrode sites that showed greater engagement for abstract words included the posterior IFG and STG (S3 Fig); other key nodes in the core language network. The pMTG abstract-preference dovetails with recent meta-analytic neuroimaging evidence that bilateral pMTG supports greater semantic control for abstract concepts [75].

Most regions in our study align with classical language areas, outside of primary sensorimotor areas, potentially indicating that the semantic organization of concreteness extends far beyond the representations invoked by embodied frameworks [71,76]. These are associated with perceptual and motor features of the denoted entity and are assumed to elicit related sensory/perceptual features [32,77–80]. Our cortical cascade model can accommodate this broad range of cross-cortical conceptual features being integrated into a lexical representation, not being limited to regional hubs common to embodied accounts. Nevertheless, given scant coverage over primary sensorimotor cortex, we are unable to adjudicate some more nuanced predictions of these theories. In addition, we note that these forms of embodied and sensorimotor reactivation accounts do find some support in certain parts of our results. Consider that concrete word effects may partially reflect reactivation of sensory features, even if these are not strictly triggered by motor or primary sensory cortices. Parahippocampal and fusiform cortices have been linked to perceptual memory [81]. As such, while our empirical coverage limits direct assessment of primary sensorimotor contributions, the presence of some ventrotemporal effects may genuinely reflect reactivation of perceptual feature information rather than lexico-semantic information alone. Indeed, these two possibilities are by no means mutually exclusive, at least with respect to a narrow focus on ventrotemporal activation profiles.

Numerous studies have revealed that abstract concepts activate IFG and bilateral MTG, while concrete concepts lead to greater activation in posterior cingulate, precuneus, angular gyrus, fusiform gyrus, and PHG [22,82,83], which largely overlaps with our semantic network, although with reverse findings attributable to the IFG. Nevertheless, we note that posterior IFG hosted a number of electrodes that showed greater activation for abstract words (S3 Fig). Prior studies have not assessed concreteness judgements but rather involved recall tasks from word lists, lexical decisions, complex sentence processing, or forms of executive control to dissociate concrete from abstract concepts [33,84]. As such, IFG may indeed be involved in the processing of abstract words (Fig 3 shows clear engagement for abstract word processing), but is generally more active during concrete concepts, at least in the case of task instructions explicitly asking participants to press “Yes” when the word *is* concrete, rather than when it is abstract. In other words, some of our documented effects could be reflections of action policy prosecution—though we highlight that many features of our semantic network were well established considerably prior to the button press. Frontal BGA increases are of course not uniquely semantic; in intracranial work they also mark domain-general cognitive control [44]. Relatedly, we note that future work could more carefully model related semantic features like imageability, age-of-acquisition, valence and contextual diversity, which all covary with concreteness.

Finally, our connectivity analysis is concordant with a study presenting subjects with concrete/abstract imagery relating to various animal descriptions, in which neural connectivity during concrete imagery was significantly higher than during processing abstract imagery [85]. Importantly, our model differs from these existing accounts in that it emphasizes the progressive and frequency-specific directed integration across cortical pathways of representations of distinct conceptual formats, in contrast to other proposals [86]. The structure of cortical cascades documented here may therefore help account for the inherent ambiguity of midscale words and the (late) temporal profile of their distinguishability in frontal sites.

### Concreteness within the semantic network

Pathologies of anterior temporal regions, such as semantic dementia, can result in a “reverse concreteness effect” where semantic memory of concrete concepts is impaired but abstract concepts are recalled more easily [87–92]. This is partially concordant with our stimulation findings that disruption of ventrotemporal cortex and frontal cortex impairs the ability to make semantic judgements about concreteness. Our work extends previous stimulation research in frontal cortex [84,93], with portions of OFC, inferior frontal cortex, and ventrotemporal cortex being causally implicated in semantic judgements.

We have previously shown that the anterior ventrotemporal cortex, encompassing regions we label here as mFus, classically referred to as the basal temporal language area (BTLA) [94], is a critical lexico-semantic hub, engaged in both visually-cued and auditorily-cued naming [95,96]. It is the earliest region sensitive to word frequency and lexicality during reading [51,97], and is also engaged in more general semantic processes [82]. Stimulation or lesioning of BTLA results in disruption of the ability to read and name [20,98,99]. Our current recordings and stimulation results provide further evidence for this region’s role in lexical semantics.

The semantic network is broadly distributed across the brain [62], and here we show a similar broad, interactive frontotemporal network recruited for semantic concreteness judgements. We observed ventrotemporal information flows to frontal regions and TPJ which were greater for concrete over abstract words, and information flows from within multiple frontal and temporal regions to OFC, which were also greater for concrete words. Our distributed PDC network resonates somewhat with more recent hub-and-spoke frameworks emphasizing dynamic frontotemporal integration rather than a single amodal hub [100], and our cortical cascade proposal adds more specific details concerning the apparent location of activation timings of these sites.

Nevertheless, we wish to stress that our limited sample size for this causal evidence precludes any stronger conclusions being drawn with respect to the localization of semantic inferences in ventral temporal and inferior frontal cortices. Although the directionality of effects was statistically significant in our stimulation cohort, we highlight the need for further replication and extension of these findings in future work. We also stress some nuances to the nature of the “causal” evidence we offer. Firstly, it is never the case that a *lack* of stimulation effect means that a stimulated region is not recruited in a certain task demand, since other brain regions can often some to the assistance of a specific computation [12]. Secondly (and conversely), the *presence* of a stimulation effect over a given brain region does not necessitate the conclusion that this brain region is causally involved in one specific computation of interest to researchers, since disrupting some ancillary function may *also* lead to disruptions for wholly independent reasons [14,15]. In the context of our study, this implies that our documented task disruptions may be driven not exclusively by conceptual processing, but by certain executive demands not specific to language or semantics. Indeed, any semantic deficit induced may also have been triggered by disruption not specifically to the stimulated region, but to a closely connected downstream region (for related discussion, see [48]). Therefore, we expect that more fine-grained stimulation parameters over more temporally acute phases of word processing will offer clearer results.

### Midscale words

If midscale words exhibit a stable conceptual representation, blending both concrete and abstract elements, regardless of patient response, then we would expect to see no differences between midscale words that were rated by patients as concrete, and midscale words rated as abstract, and this is indeed what we discovered. Despite some inherent limitations to our statistical tests (see below), we believe that our results expose an invariant neural signature for conceptual features that compose into lexical items, independent of human behavior. Considering our stimulation results, the causal role of OFC may be attributed more to the coding of salient semantic features for the task, given that it showed midscale rating sensitivity and given also the place of ventral prefrontal cortex in default-mode activity [101]. In contrast, ventrotemporal sites with a causal role in concreteness judgements may be more directly involved in specific lexico-semantic features, and not only task-related semantic salience, given that ventrotemporal sites showed no difference based on midscale ratings. Interestingly, participants had an abstractness bias for the majority of midscale words (65 ± 18% of midscale words were rated as abstract). We note that we found no difference in response time between midscale (mean: 989 ms) and abstract (mean: 986 ms) words, suggesting that our potentially difficult task design (i.e., instructing participants to choose between concrete and abstract, while presenting them with midscale words) likely did not impact substantial elements of semantic processing. Nevertheless, future work could explore which factors may be driving sense selection in midscale words (e.g., sense frequency, essentialism, prototypicality, polysemy type) and whether these factors impact neural concreteness signatures, potentially across distinct frequency bands.

Yet this raises a question: What precisely are the features that gamma signatures are indexing? Midscale words used in our study ranged across “royalty,” “district,” “doctorate,” “corporation,” “translation,” and “magic” [42], but what features does a word need to have in order for it to be likely rated in the middle of the concreteness scale? Many words that have typically been judged by participants as midscale are simply those words about which participants tend to disagree [42]. From the perspective of polysemy, many of these words can jointly host *both* concrete and abstract senses (e.g., a “translation” can be an abstract process, or a physical document; a “corporation” can be sued, but also built next to a library). Psycholinguistic research has recently come to the general assessment that these types of broadly polysemous words, when accessed in isolation, trigger an underspecified representation of the word that hosts features of all semantically related senses, thus allowing easier processing and representational dedication upon later decision-making stages [37,102–104]. As such, any differences between midscale words based on participant subjective ratings would be expected to occur at late processing stages. This prediction was satisfied by our results (Fig 4), suggesting that there is some invariant, underlying representation that is being accessed, regardless of how patients are individually assessing the concreteness of midscale words. Indeed, the responses of midscale words did not significantly deviate from the expectation of being an intermediary between concrete and abstract words, with no unique responses relating to being near the decision boundary (S2 Fig).

One alternative interpretation to our midscale results would be to suggest that participants were randomly choosing their responses when exposed to midscale words that appeared ambiguous to them. However, this randomness hypothesis is not supported by the abstract-leaning bias of midscale ratings, in addition to the similar response times (if patients struggled with inherent ambiguity before randomly selecting a response, response times would be longer for midscale words). In addition, each of the midscale words still afforded a salient reading from either a concrete or abstract perspective, and so participants likely made judgments based on whichever sense they arrived at first (i.e., “profit” may trigger the concrete physical money sense for some participants, but other participants may be more biased towards the abstract quantity reading).

It is also of interest here that the parsing of bistable perceptual states recruits inferior frontal areas [105]. In our tasks, midscale words represented a not dissimilar abstract representation, with participants’ judgments about these words occurring alongside sensitivity to concreteness in OFC and FO (Fig 4). There may some overlap here in terms of decision-making networks being recruited to resolve inherently ambiguous representations.

There is an additional issue concerning the representation of linguistic context (whereby concrete/abstract words can be placed in different narrative contexts), which has recently posed a challenge to the notion that there are dedicated cortical circuits for concreteness [106]. While this domain demands further exploration, we note that our cortical stimulation mapping, our semantic network, and our results concerning the rating-independent signatures for midscale words, all indicate the likely existence of some context-invariant semantic structures that can be accessed by distinct network nodes.

### Limitations

We wish to highlight here an important limitation concerning the interpretation of the SVR comparison between the response-based and midscale models. The midscale model predicts a constant concreteness value (3) for all midscale trials, whereas the response-based model predicts values that vary between 1 and 5 depending on participants’ behavioral classifications. This creates an intrinsic variance asymmetry between the two competing label sets. Because mean squared error is sensitive to label variance, comparisons against a constant intermediate label may mathematically favor the midscale model, even if neural responses partially track subjective decisions. Consequently, the lower MSE observed for the midscale model should not be interpreted as definitive evidence that neural representations of midscale words are independent of subjective judgement. Instead, this analysis should be interpreted cautiously and in conjunction with other empirical observations reported here. These include the absence of reliable broadband gamma differences between midscale trials rated as concrete versus abstract across most regions of interest across most ROIs. Future work could address this limitation using alternative analytical strategies that control for label variance. Such approaches may help more precisely dissociate conceptual representational structure from decision-related variability.

Lastly, a key concern of human intracranial EEG studies is the extent to which findings can be extrapolated to the general population. Participants had no history of prominent language deficits, and we have previously shown that activations in the brains of individuals with epilepsy are not dissimilar compared to healthy volunteers across semantic classes that bear some general similarity to our present concerns (nouns and verbs) [107]. Nevertheless, we acknowledge that effects of epilepsy and possible secondary reorganization of brain regions outside diagnosed seizure foci (diaschisis), in addition to potential longer-term effects of antiseizure medications, are common to all human intracranial EEG studies [108,109], and remain unavoidable features of our work.

### Conclusions

We provide a directed, temporally ordered, and causally validated account of how written words are used to derive conceptual judgments. We discovered that rapid semantic judgements activated a distributed but tightly interconnected frontotemporal network via cascades of information flow. Crucially, our results support a stable and invariant representational code: midscale words elicit essentially the same activation patterns regardless of trial-by-trial classification. The earliest sites of semantic sensitivity were in ventrotemporal cortex, and later effects were found in lateral frontal and posterior temporal cortex. Posterior middle temporal cortex showed greater engagement for abstract words, pointing to this region’s previously implicated involvement in structured symbolic meaning. We utilized cortical stimulation mapping in five patients to attribute causal involvement of ventrotemporal cortex and inferior frontal cortex by disrupting their ability to accurately make concreteness judgements. These results add important causal weight to our documented semantic network and model, and we expect that these cortical dynamics contribute in fundamental ways to the neural basis of conceptual knowledge.

## Materials and methods

### Participants

Nineteen patients (6 male, 19–47 years, all right-handed, IQ 96 ± 8, Age of Epilepsy Onset 20 ± 11 years) participated in the experiments after written informed consent was obtained. All participants were semi-chronically implanted with intracranial electrodes for seizure localization of pharmaco-resistant epilepsy. All were native English speakers. Participants were excluded if they had confirmed right hemisphere language dominance, if they only had electrode coverage of the right hemisphere, or if they had a significant additional neurological history (e.g., previous resections, MR imaging abnormalities such as malformations or hypoplasia). All patients underwent extensive presurgical neuropsychological assessments and we only included individuals able to perform the task within acceptable performance characteristics. Three additional participants performed the task but were excluded from analysis as their behavioral performance in the concreteness judgement task was not significantly above chance. All experimental procedures were reviewed and approved by the Committee for the Protection of Human Subjects (CPHS) of the University of Texas Health Science Center at Houston as Protocol Number HSC-MS-06–0385.

### Electrode implantation and data recording

Data were acquired from either subdural grid electrodes (SDEs; 1 patient) or stereotactically placed depth electrodes (sEEGs; 18 patients). SDEs were subdural platinum-iridium electrodes embedded in a silicone elastomer sheet (PMT Corporation; top-hat design; 3 mm diameter cortical contact), and were surgically implanted via a craniotomy [46,110,111]. sEEGs were implanted using a Robotic Surgical Assistant (ROSA; Medtech, Montpellier, France) [112,113]. Each sEEG probe (PMT corporation, Chanhassen, Minnesota) was 0.8 mm in diameter and had 8–16 electrode contacts. Each contact was a platinum-iridium cylinder, 2.0 mm in length and separated from the adjacent contact by 1.5–2.43 mm. Following surgical implantation, electrodes were localized by co-registration of pre-operative anatomical 3T MRI and post-operative CT scans in AFNI [114]. Electrode positions were projected onto a cortical surface model generated in FreeSurfer [115], and displayed on the cortical surface model for visualization [110]. Intracranial data were collected during research experiments starting on the first day after electrode implantation for sEEGs and two days after implantation for SDEs. Data were digitized at 2 kHz using the NeuroPort recording system (Blackrock Microsystems, Salt Lake City, Utah), imported into MATLAB, initially referenced to the white matter channel used as a reference for the clinical acquisition system and visually inspected for line noise, artifacts and epileptiform activity. Electrodes with excessive line noise or localized to sites of seizure onset were excluded. Each electrode was re-referenced to the common average of the remaining channels. Trials contaminated by inter-ictal epileptic spikes, saccade artifacts, and trials in which participants responded incorrectly were discarded.

### Experimental design and statistical analysis

All participants undertook a concreteness judgement task during passive recording, with five of these patients also performing a modified version of this task (optimized for the demands of stimulation mapping) during direct cortical stimulation.

### Concreteness judgement task

Patients were presented with single words on a screen and asked to press a button indicating whether or not the word referred to something you could touch, taste, smell, or hear (Fig 1A). Stimuli were presented using Psychtoolbox [116] on a 15.4” LCD screen positioned at eye-level, ~60 cm from the patient (white screen, black Arial font, 120 pixel height, ~1.8° visual angle). Each word was presented for 1,000 ms, followed by a fixation cross for 1,500 ms. Participants responded by pressing either the left arrow (“Concrete”) or right arrow (“Abstract”). This task minimized working memory demands, with patients being able to respond as soon as they had made their judgement. Participants were presented with 80 trials each of concrete, abstract, and midscale words in a pseudorandom order (240 trials total). The full experiment lasted approximately 10 min and was completed in a single block with no breaks.

Concrete, abstract, and midscale stimuli were taken from a previous study that carefully selected items based on their concreteness ratings from a number of independent databases. Concrete and abstract words were selected that showed high agreement between studies and individuals, and with midscale items displaying mean concreteness ratings occupying the middle range of the 1–5 Likert scale and exhibiting larger standard deviations (i.e., exhibiting greater inter-individual disagreement) [42,43]. Properties of the stimuli are summarized in S1 Fig, and in Table 1 we provide additional summary information. Emotion words were omitted (i.e., any item that could be readily inferred as denoting a subjective emotional state, like “frustration”), given that these are often considered in the literature to be somewhat concrete and abstract, with many theorists holding that “affective” states should be considered as concrete as traditional sensory states [117–119].

**Table 1:** Summary information for word stimuli.

	Mean concreteness	SD concreteness	Age of acquisition	Zipf frequency	Syllable number	Letter number	Mean bigram frequency	Absolute emotional valence	% known words
**Concrete**	4.55 (0.17)	0.81 (0.12)	10.11	3.41 (0.48)	2.42 (0.86)	7.63 (1.79)	3,649 (1,134)	1.12 (0.77)	99
**Abstract**	1.61 (0.15)	0.85 (0.11)	10.2 (1.95)	3.54 (0.72)	2.53 (0.89)	7.63 (1.95)	3,710 (1,208)	1.15 (0.78)	99
**Midscale**	3.02 (0.26)	1.51 (0.77)	10.11 (1.99)	3.53 (0.72)	2.54 (0.86)	7.57 (1.89)	3,737 (1,184)	1.15 (0.77)	98.7

### Cortical stimulation

In five patients, we performed chronometric stimulation, time-locked to the onset of the presented word and lasting for the 1-s duration the word was on the screen. For each presented word, there was a 50% chance of stimulation being applied, with patients blinded to whether stimulation was being applied on a given trial. Only concrete and abstract words were used, that the participants had correctly assigned to the expected category in the previous task (in contrast to the button press of the prior intracranial task). Trial onsets were manually triggered by the experimenter and participants gave their responses verbally. Cortical stimulation was carried out through neighboring bipolar electrode pairs using 500 µs wide, constant-current, square waves, delivered at 50 Hz, in a biphasic manner to prevent charge deposition, with current amplitudes between 4 and 10 mA (safely established during an immediately preceding clinical stimulation mapping session). Stimulation was performed using a Grass S88X stimulator (Grass Technologies, West Warwick, RI) or a Blackrock CereStim (Blackrock Neurotech, Salt Lake City, UT). Inclusion criteria were (i) good performance on the concreteness task, and (ii) electrode coverage over one of the major ROIs evinced from our grouped analyses (ventral temporal, inferior frontal).

### Signal analysis

Analyses were performed by first bandpass filtering the raw data of each electrode into broadband gamma activity (BGA; 70–150 Hz) following removal of line noise and its harmonics (zero-phase 2nd order Butterworth band-stop filters). Electrodes were also visually inspected for saccade artifacts. A frequency domain bandpass Hilbert transform (paired sigmoid flanks with half-width 1.5 Hz) was applied and the analytic amplitude was smoothed (Savitzky-Golay FIR, 3rd order, frame length of 201 ms; MATLAB 2020b, Mathworks, Natick, MA). BGA was defined as percentage change from baseline level: −500 to −100ms before the presentation of the first word in each trial. Electrodes were considered responsive if they showed mean BGA > 10% in the time window 300–700 ms after word onset, based on prior intracranial studies of reading [51,62]. Of the 2,241 electrodes located in left, language-dominant cortex, 1,030 electrodes (in 19 patients) were considered responsive (Fig 1D).

ROIs were selected in areas identified in previous intracranial studies of reading [50,51,97,120] and areas from the surface-based analysis. ROI centers were defined on the cortical surface and all responsive electrodes within a set geodesic radius of this point were included [121]. This method was selected as currently available cortical surface parcellations have been shown to be inadequate at predicting functional boundaries of task-evoked activity [122]. Centers of mass for each of the ROIs, in Talairach space, were as follows: mFus, −32 −35 −17; PHG, −25 −16 −22; TPJ, −49–52 25; pIFG, −42 15 30; aIFG, −39 33 18; FO, −38 17 9; OFC, −34 26–8.

Linear mixed effects (LME) models were used to dissociate multiple factors modulating BGA over time. The primary fixed effect used was concreteness, with word length and word frequency included as regressors of no interest. Hierarchical random effects were used, with the random effect of individual electrodes grouped by patient. For example, the following model definition was used to probe the effects of concreteness on BGA within an ROI: *BGA ~ Concrete + WordLength + WordFrequency + (1|Patient) + (1|Patient:Electrode)*. Frequentist statistical methods were corrected for multiple comparisons using a Benjamini–Hochberg false detection rate (FDR) correction (*q* < 0.01). For the grouped analysis, all electrodes were averaged within each subject and then the between-subject averages were used.

### Surface-based linear mixed effects (sbLME) modeling

sbLME [50] was used to map electrode activations mapped onto the standardized population brain surface using each electrode’s presumed “recording zone,” an exponentially decaying geodesic radius [112,123]. LME models were then used at each vertex of the standardized surface. Results were thresholded at a t-statistic greater than 2 and coverage of at least 3 patients. Cluster significance was computed at a corrected alpha-level of 0.01 using family-wise error rate corrections for multiple comparisons. The minimum criterion for family-wise error rates was determined by white-noise clustering analysis (Monte Carlo simulations, 1,000 iterations) of data with the same dimension and smoothness as that analyzed [123].

### Neural decoding

Decoding analyses were performed using linear SVM and SVR classifiers. Concreteness decoders used 10-fold cross-validation, and the midscale decoders used independent test and training sets, being trained on data from the concrete and abstract trials and tested on the midscale trials. For each patient, decoding performance was summarized with an area under the curve (AUC) value. Temporal decoding was performed on BGA using a sliding estimator at each time point, using all available electrodes. Statistical significance against chance was tested using a null distribution, training 1,000 decoders using randomly shuffled condition labels.

### Partial directed coherence (PDC)

PDC [124] was calculated using patient-specific multivariate time series, fit to a predictive multivariate autoregressive (MVAR) model on each window [125]. We note the typical caveat here that PDC does not indicate “true” causation but instead suggests directed statistical dependence. PDC was calculated pair-wise for each pair of electrodes across ROIs, within-individual. Hilbert-transformed, gamma-band envelope (70–150 Hz) data, aligned to stimulus onset, and divided into overlapping windows (length 100 ms, shift 50 ms) were used. A model order of three was used resulting in a frame length of 300 ms. Mean PDC from 500 to 800 ms was fit using LME models, for each ROI pair, with a fixed effect for concreteness, and a hierarchical random effect of electrode pair, grouped by patient. Indegree and outdegree centrality were calculated, for the intercept and for positive concreteness effects, as the weighted sum of inputs/outputs for each node of the network. Confidence intervals for the centrality metrics were generated by bootstrapping 500 networks, randomly resampling with replacement within each inter-ROI connection.

### Inclusion and ethics

Participants provided written informed consent. All procedures performed in this study involving human participants were conducted in accordance with the principles expressed in the Declaration of Helsinki. All experimental procedures were reviewed and approved by the Committee for the Protection of Human Subjects (CPHS) of the University of Texas Health Science Center at Houston as Protocol Number HSC-MS-06-0385.

## Supporting information

S1 Fig Lexical statistics for the stimulus set. Distribution of **(A)** mean and **(B)** inter-individual standard deviation of concreteness ratings from a large population behavioral study [112], **(C)** word frequency [113], and **(D)** word length in number of letters. Source data for these results can be found at OSF (osf.io/jyvg2/).
(S1_Fig.TIF)

S2 Fig Surface-based dissociation of midscale words. Surface-based linear mixed effects (sbLME) modeling including regressors for **(A)** concreteness, with concrete, midscale and abstract words on an ordinal scale, with **(B)** a specific regressor for midscale words. This analysis was designed to seek regions where midscale words deviate from the expected mean concreteness hierarchy.
(S2_Fig.TIF)

S3 Fig Surface-based dissociation of concrete versus abstract words. **(A)** Surface-based linear mixed effects (sbLME) model from Fig 3B plotted with a lower t-threshold (*t* > 1, coverage > 2 patients, *p* < 0.01 corrected). **(B)** Single electrode contrasts of concrete versus abstract trials showing all electrodes with an absolute difference of Δ%BGA > 10. **(C)** sbLME map of the effect of concreteness in the pre-response period (−250 to 0 ms; *t* > 2.5, coverage > 2 patients, *p* < 0.01 corrected).
(S3_Fig.TIF)

## Abbreviations

AUC: area under the curve
BGA: broadband gamma activity
BTLA: basal temporal language area
FDR: false detection rate
FO: frontal operculum
IFG: inferior frontal gyrus
LME: linear mixed effects
mFus: mid-fusiform cortex
MTG: middle temporal gyrus
OFC: orbitofrontal cortex
PHG: parahippocampal gyrus
pMTG: posterior middle temporal gyrus
sbLME: surface-based linear mixed effects
SDEs: subdural grid electrodes
sEEGs: stereotactically placed depth electrodes
SVM: support vector machine
SVR: support vector regression
TPJ: temporoparietal cortex
