# Supplementary figures and images for: Frontotemporal network interactions causally support rapid concreteness judgments during reading
Source: PLoS Biol. 2026 Mar 25;24(3):e3003723. doi: 10.1371/journal.pbio.3003723 (PMC13016318; doi:10.1371/journal.pbio.3003723)

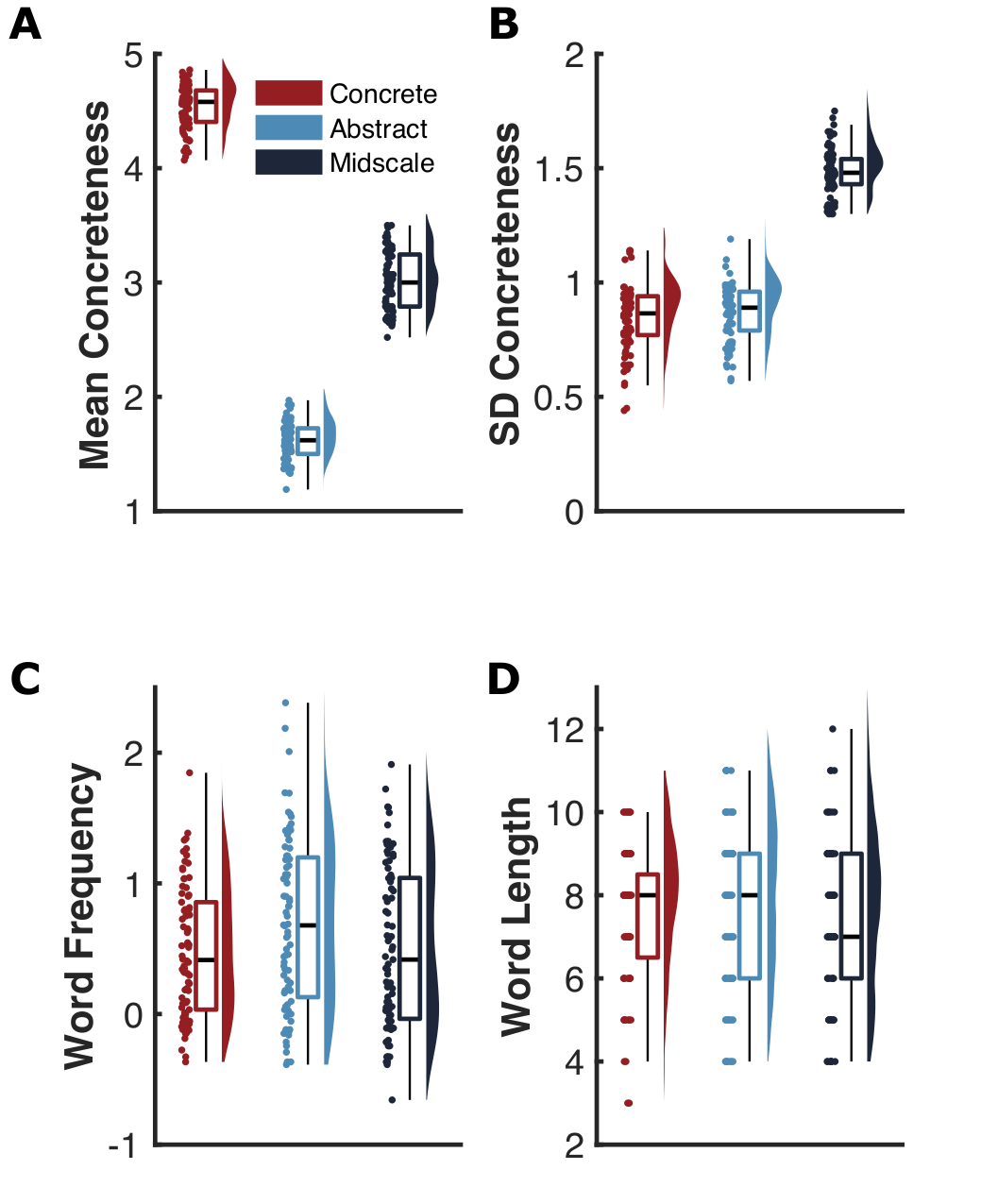

Supplement: S1 Fig — Distribution of (A) mean and (B) inter-individual standard deviation of concreteness ratings from a large population behavioral study [112], (C) word frequency [113], and (D) word length in number of letters. Source data for these results can be found at OSF (osf.io/jyvg2/). (S1_Fig.TIF) [file pbio.3003723.s001.tif]

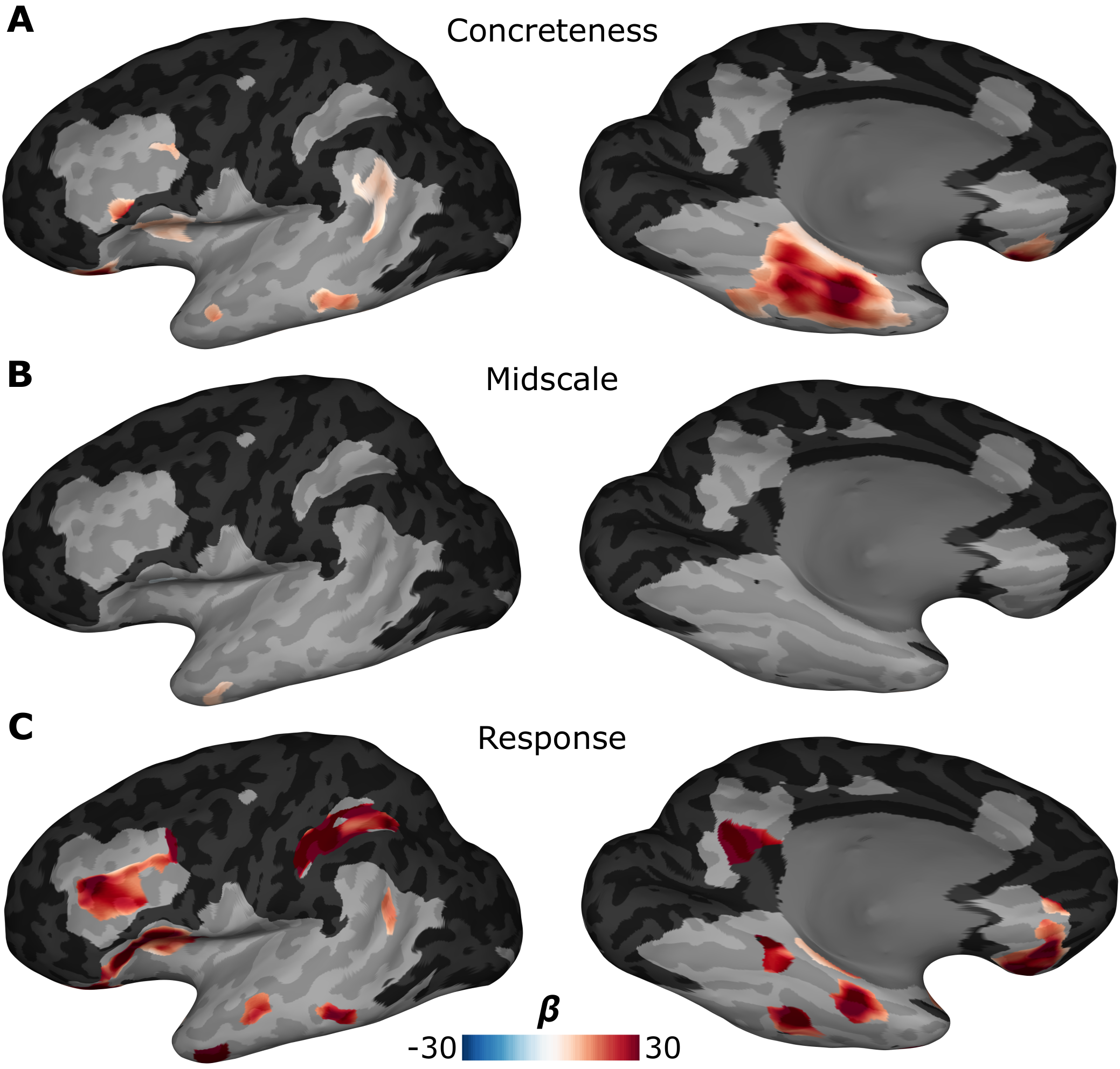

Supplement: S2 Fig — Surface-based linear mixed effects (sbLME) modeling including regressors for (A) concreteness, with concrete, midscale and abstract words on an ordinal scale, with (B) a specific regressor for midscale words. This analysis was designed to seek regions where midscale words deviate from the expected mean concreteness hierarchy. (S2_Fig.TIF) [file pbio.3003723.s002.tif]

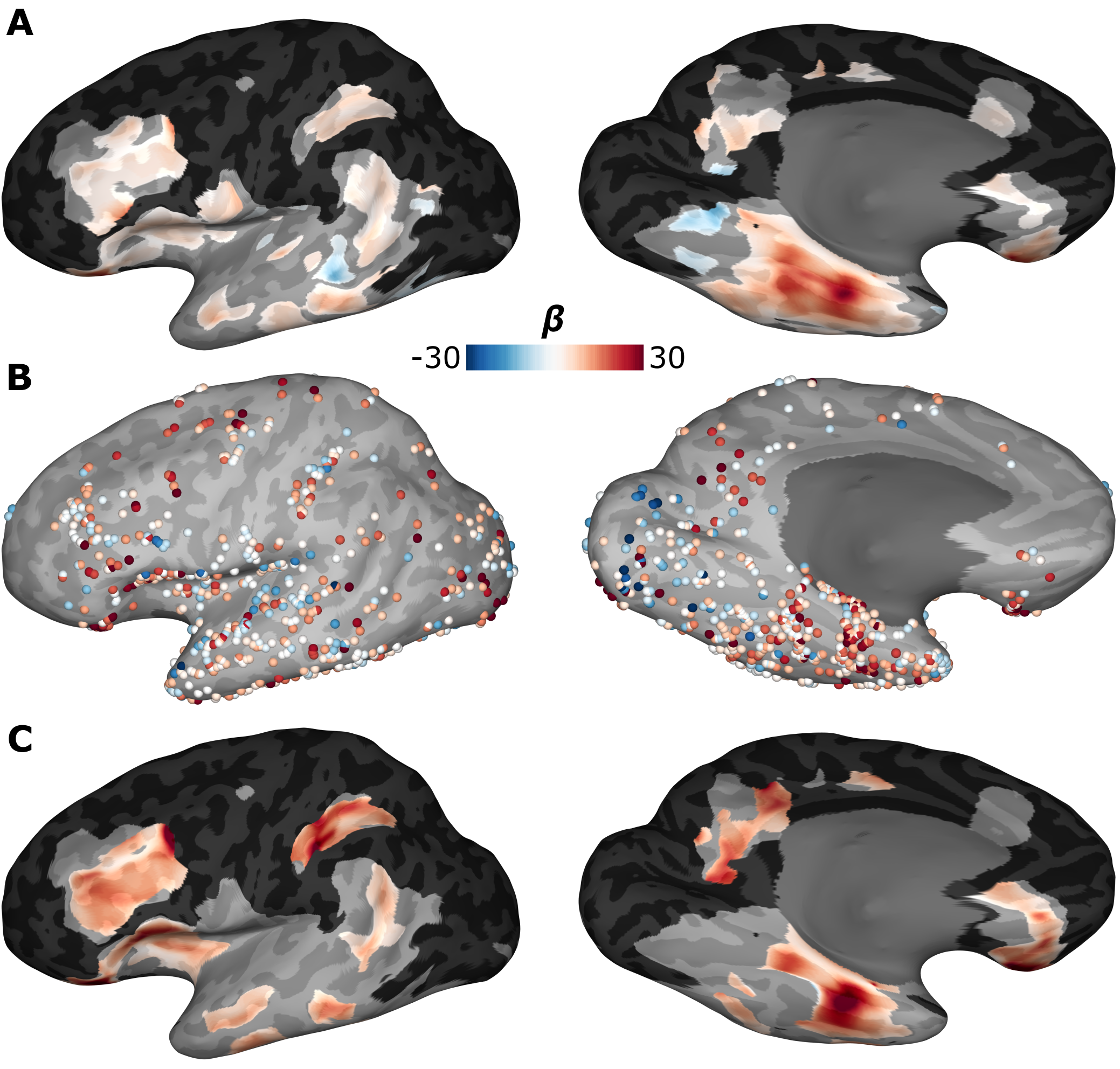

Supplement: S3 Fig — (A) Surface-based linear mixed effects (sbLME) model from Fig 3B plotted with a lower t-threshold (t > 1, coverage > 2 patients, p < 0.01 corrected). (B) Single electrode contrasts of concrete versus abstract trials showing all electrodes with an absolute difference of Δ%BGA > 10. (C) sbLME map of the effect of concreteness in the pre-response period (−250 to 0 ms; t > 2.5, coverage > 2 patients, p < 0.01 corrected). (S3_Fig.TIF) [file pbio.3003723.s003.tif]
